# The Structural Framework and Opening Appearance of the VP1-Pocket of Enteroviruses Correlated with Viral Thermostability

**DOI:** 10.3390/pathogens13080711

**Published:** 2024-08-22

**Authors:** Xiaojing Lin, Jianhong Gan, Qiang Sun, Zi Li, Kun Qin, Yong Zhang, Yang Cao, Jianfang Zhou

**Affiliations:** 1 National Key Laboratory of Intelligent Tracking and Forecasting for Infectious Diseases (NITFID), National Institute for Viral Disease Control and Prevention, Chinese Center for Disease Control and Prevention, Beijing 102206, China; lin.alpha@foxmail.com (X.L.); sunqiang8611@126.com (Q.S.); lizi@ivdc.chinacdc.cn (Z.L.); qinkun@ivdc.chinacdc.cn (K.Q.); zhangyong8@ivdc.chinacdc.cn (Y.Z.); 2 Center of Growth, Metabolism and Aging, Key Laboratory of Bio-Resource and Eco-Environment, Ministry of Education, College of Life Sciences, Sichuan University, Chengdu 610064, China; gan_jianhong@stu.scu.edu.cn (J.G.); caoyang11123@163.com (Y.C.)

**Keywords:** thermostability, enteroviruses, VP1-pocket, pocket opening

## Abstract

Enteroviruses (EVs and RVs) are prevalent worldwide and cause various diseases in humans, of which the VP1-pocket is a target of antivirals, with a lipid molecule as a pocket factor to stabilize the virion. However, the characterization of the structure of the VP1-pocket in EVs is poor. Here, we compared the published capsid crystals of EVs and RVs and proposed a structural framework for the VP1-pocket: Frame 1–4, which is located at the CD loop, GH loop, and C-terminus, presenting with an outward opening appearance or not. The non-outward viral strains—CVB3, Echo 11, RV-A81, and RV-B70—are more thermally stable, with a breakpoint temperature (B.T.) of 51~62 °C for genome releasing, which is 4~10 °C higher than its outward temperature of 41~47 °C, and infectivity preservation when treated at 50 °C for 3 min. Its outward versus non-outward opening is correlated significantly with the B.T. for genome release (*r* = −0.90; *p* = 0.0004) and infectivity (*r* = −0.82, *p* = 0.0039). The energy of Frames 1, 2, and 4, including *Van der Waals* attractive and repulsive interactions and hydrogen bonds, showed significant correlations with the B.T. (r = −0.67, 0.75, and −0.8; *p* = 0.034, 0.013, and 0.006, respectively). These characters of the VP1-pocket could be predictors for virion thermostability and aid in the development of vaccines or antivirals.

## 1. Introduction

Picornaviruses have a large number of members with single, positive-stranded RNA and an icosahedral capsid of approximately 30 nm diameter, of which the Enterovirus genus has caused a wide range of human diseases both historically and currently, from mild to fatal, including the enterovirus (EV) A–D and rhinovirus (RV) A–C species [1,2]. These non-enveloped viruses are more stable with the protection of a icosahedral capsid. Many EVs are food and environmental contaminants, which can survive in the matrix of high salt and heat conditions and infect humans via fecal–oral transmission [3]. Notably, to promote vaccine coverage against EVs, developing a thermally stable vaccine is a priority [4].

The viral capsid of EVs and RVs possesses 12 pentamers, which are formed by five repeating protomers constructed by VP1–4. VP1–3 are located on the surface of the virion, with eight antiparallel β-strands named by the letters B to I and the loops named using two letters designating the β-strands that the loop connects [5]. In each pentamer, five antiparallel β-barrels of VP1 form a protrusion termed the 5-fold axis, with a deep encircling depression called the canyon that also frequently serves as the receptor binding site [6]. This antiparallel β-barrel and the junction loops of VP1 form a hydrophobic pocket that is occupied by a lipid molecule, namely the pocket factor, which is considered to stabilize the 160S mature particle naturally [7].

Once bound to an uncoating receptor, the particles switch into a 135S A-particle irreversibly. With the insertion of the N terminus of VP1, forming a pore in the membrane through which the RNA could be extruded into the cytoplasm, an 80S empty particle is formed [6,7]. Based on the universal properties of the pocket, capsid binders—a kind of small molecule that competes with the natural pocket factor with a higher affinity to the VP1-pocket to inhibit uncoating—were developed [8]. The capsid binders could enhance the virion’s thermostability and hinder genome release by supporting the pocket with its rigid structure, maintaining the capsid’s conformation [8,9]. Moreover, VP1-V87A and VP1-I194V were detected in several heat-resistant variants of type 1 Poliovirus, and these VP1s formed a deeper or more spacious pocket, accommodating the pocket factor at up to 51 °C [10].

Thermostability as a determinant factor for vaccine development is well-recognized. Nevertheless, the thermal profiles of different species or types of EVs and the underlying molecular mechanisms remain unknown. Here, we profiled the features of the VP1-pocket, proposed a framework for it, and discovered the correlation between its opening appearance and viral thermostability using ten representative strains covering EV A–D and RV A–B, which may provide insights into the current understanding of the VP1-pocket.

## 2. Materials and Methods

### 2.1. Cells and Viruses

Rhabdomyosarcoma (RD) cells and H1-Hela cells were obtained from the American Type Culture Collection. RD cells were cultured in Dulbecco’s modified Eagle’s medium (DMEM; Invitrogen, Carlsbad, CA, USA), supplemented with 10% fetal bovine serum (FBS, Gibco, Grand Island, NY, USA). EVs and RVs were maintained or isolated in our laboratory, including EV-A71/SZK2021, CVA6/BJ103, CVA10/HeB2020-063, Echo30/WZ16, Echo11/HeB2017-231, CVB3/K22156T, Poliovirus3/nOPV3, EV-D68/21286, RV-A81/bj1295, and RV-B70/bj1777. EVs were propagated in RD cells and RVs were propagated in H1-Hela cells, supplemented with 2% FBS in DMEM. The virus titer was measured by the observation of cytopathogenic effect (CPE) after 24 h incubation in RD or H1-Hela cells. The tissue culture infectious dose affecting 50% of the cultures (TCID_50_) was calculated by the Reed–Muench formula. The concentrated virions were prepared from 200 mL high-titer virus-infected cell lysate, frozen and thawed twice and the cell debris removed by centrifugation at 4000 rpm for 120 min. The supernatant was mixed with 10% PEG8000 (Sigma-Aldrich, Saint Louis, MO, USA) and 0.5 M NaCl, kept at 2–8 °C overnight, then centrifuged at 4000 rpm for 120 min; the pellets were reconstituted in 5 mL PBS, then 1250U Super Nuclease (Beyotime, Shanghai, China) was added and digested at 37 °C for 1 h. After digestion, 5 mL of trichloromethane was added and mixed vigorously. The mixture was centrifuged at 4000 rpm for 60 min and the aqueous solution was collected and this step repeated once. The final aqueous solution was further concentrated by ultrafiltration of 30K Ultra-15 mL at 4000 rpm for 30–60 min to obtain an about 300–500 μL virion concentration.

### 2.2. Infectivity-Based Thermostability Assay 

Infectivity-based thermostability was conducted using a PCR thermocycler. EVs and RVs (10^4^–10^5^TCID_50_/50 μL) were incubated at 37 °C for 5 min and then 50 °C for 3 min, followed by rapid cooling to 4 °C. Subsequently, 23 μL of the mixture was diluted by 50 μL DMEM to get a continuous 0.5 Log_10_ dilution and was added to RD or H1-Hela cells. After 24 h infection, the CPE was observed, stained by crystal violet. Virus titers were determined in terms of the mean TCID_50_ per 50 μL via the detection of CPE in cells.

### 2.3. Fluorescence-Based Viral Genome Release Assay 

A 20 μL reaction mixture was set with 2 μL of 50X Sybr green II dye (diluted from 10,000X, Solarbio, Beijing, China), 1 μL RNaseOUT (Thermo Fisher Scientific, Carlsbad, CA, USA) at a concentration of 1 U/μL, and about 4 μg concentrated virions in PBS. To detect the dynamic processes of genome release, the reaction process included direct ramping and 50 °C pre-treatment for 40 min with fluorescence detection at 0 min, 3 min, 10 min, 20 min, 30 min, and 40 min, with direct ramping after that. The direct ramping set was 37 °C for 15 min, then ramped from 37 °C to 90 °C, and lowered to 30 °C. The fluorescence intensity was recorded at 10 points of the ramp step at 1 °C intervals using a real time PCR system (Applied Biosystems Q5). The data of the fluorescence intensity were normalized and derived with respect to temperature using GraphPad Prism 9.5.1. The temperature corresponding to the maximum derivative values of the curve, denoted as the breakpoint temperature (B.T.), was used for the measurement of thermostability. The experiment was repeated in duplicate.

### 2.4. Viral Structures and Bioinformatic Analysis

Mature viral structures were selected by the VP1 evolutionary analysis of the Maximum Likelihood method and downloaded from the Protein Data Bank, https://www.rcsb.org/ (accessed on 11 March 2024), the PDB IDs of which are 8E2X (EV-A71), 7QW9 (CVA6), 6ACU (CVA10), 7C9S (Echo 30), 1H8T (Echo 11), 7VY0 (CVB3), 1PVC (Poliovirus 3), 4WM8 (EV-D68), 1ND2 (RV-A16), and 1R09 (RV-B14). Missing side chain atoms in the viral structure were repaired using CISRR [11]. To analyze the pocket factor binding energy, 3D coordinate and topology files for proteins and pocket factors were prepared using Gromacs [12] and AmberTools [13], with a force field of AMBER99SB-ILDN and ff99SB, respectively. Hydrogen atoms were reconstructed referring to the force field. Periodic simulation boxes were created with solvent water molecules using the SPC water model. Na^+^ and Cl^−^ ions were added to maintain electroneutrality and to ensure an ion concentration of 0.15 mmol/L; 3000 steps of energy minimization were performed after the system was prepared, with the steepest descent minimization. Finally, the binding energies between the pocket factor and protomer were calculated using Gromacs’ g_mmpbsa tool [14]. 

To analyze the framework peptide of the VP1-pocket, multiple sequence alignments were performed using MAFFT [15] to extract the amino acid sequences of the framework regions. Subsequently, EvoEF2 [16] was employed to calculate various energy terms for Frame 1, 2 and 4, including Van der Waals attractive forces, Van der Waals repulsive forces, Coulomb’s electrostatic interactions, and hydrogen bond energy.

### 2.5. Statistical Analysis

Data are expressed as mean ± SD. Statistical significance was determined using two-tailed one-way ANOVA in the GraphPad Prism software package (Version 9.5.1, San Diego, CA, USA). Correlations were computed using Pearson’s r and regressed linearly. A probability (*p*) value < 0.05 was considered to indicate statistical significance. * Indicates *p* < 0.05; ** indicates *p* < 0.01; *** indicates *p* < 0.001, and **** indicates *p* < 0.0001.

## 3. Results

### 3.1. The Structure of the VP1-Pocket and Its Opening 

**Figure 1:**
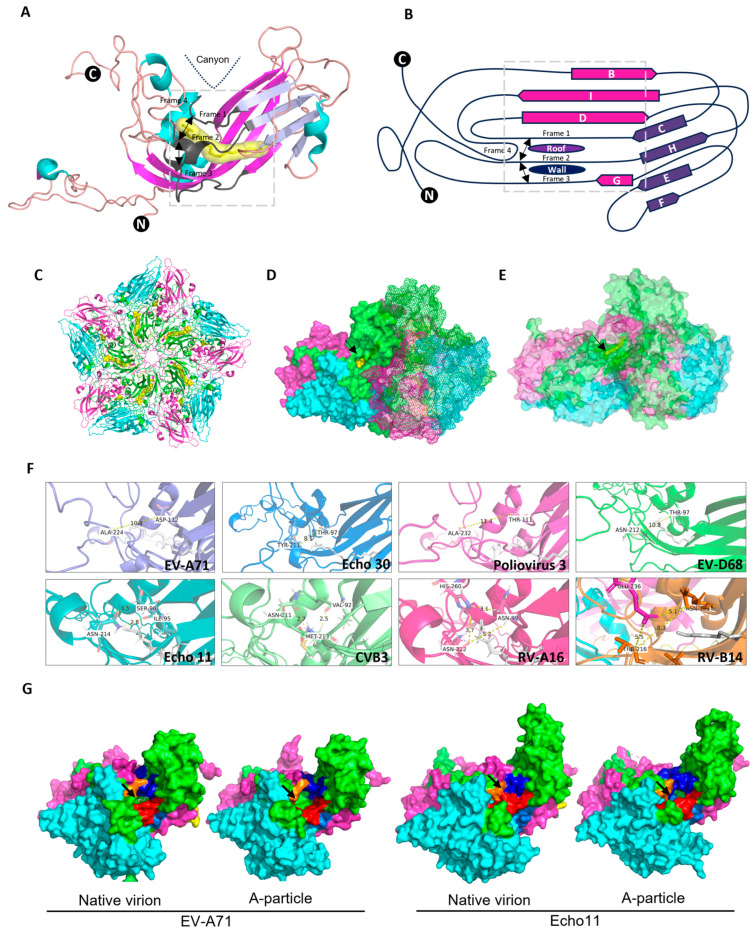
Structural analysis of the VP1-pocket opening of EVs and RVs. (**A**) Capsid protein VP1 shown in cartoon, representing the pocket factor in yellow. (**B**) Scheme of capsid protein VP1. The pocket opening-related frame located in the connection loop between CD β-strands (denoted Frame 1) and GH β-strands (denoted as Frame 2 and Frame 3), by which Frame 1 and 2 formed the roof of the pocket outwardly on the surface while Frame 2 and 3 formed the wall of pocket buried within the pentamer. The bidirectional black arrow indicated the relative positions among Frame 1, 2, and 3. (**C**) The pentamer shown in cartoon with the pocket factor in yellow. (**D**) The pocket opening on the roof unmasking the pocket factor (EV-A71). (**E**) The pocket opening not-on-the-roof masking the pocket factor (Echo11). (**F**) Maximum distance measurement between the side chains of Frame 1 and 2, for which EV-A71, Echo 30, Poliovirus3, and EV-D68 had pocket openings on the roof, and Echo 11, RV-A16, and RV-B14 had pocket openings not-on-the-roof. (**G**) Frame displacement of the VP1-pocket from native virion to A-particle; Frames 1, 2, 3, and 4 colored in blue, red, aquamarine, and orange, respectively. VP1, VP2, and VP3 colored in green, cyan, and magenta, respectively. The black arrow indicated the opening appearance.

According to the experimental enterovirus structures deposited in the Protein Data Bank (PDB), we proposed a pocket framework for the VP1-pocket that includes three frames lined along the CD loop, GH loop (Figure 1A,B), and Frame 1 near the C-terminus, with an opening on the floor of canyon (also on the roof of the pocket, formed by Frame 1, 2, and 4), and/or on the wall of the pocket (formed by Frame 2 and 3). The dissociation of the pocket factor from the virus pocket was the first step in initiating virus conformational changes when binding to the uncoating receptor; we speculated that the movement of the pocket factor might be bottlenecked by the pocket opening. Of interest, the opening of the mature particles of EV-A71 CVA6, CVA10, and EV-D68 oriented to the floor of the canyon on the surface, whereas those of Echo 11, CVB3, RV-A16, and RV-B14 directed to the wall, and those of Echo 30 and Poliovirus 3 faced both directions. In the context of a pentamer, the opening on the roof may unmask the pocket factor to the capsid surface (denoted as outward, Figure 1C,D), and the opening on the wall may be buried by another copy of the protomer when forming the pentamer (denoted as non-outward, Figure 1E). 

We then aligned the amino acid (aa) sequence of the framework. Around 9–11 aa-length residues were found in Frame 1–3 and three aa-length residues in Frame 4. Frame 1 was located between the conserved Site 109W and 118Q, Frame 2 spanned from 222Y to 231G, Frame 3 covered the residue 193P to 204F and Frame 4 ranged from 274K to 276N (EV-A71 numbered) (Figure S1). The outward opening located on the capsid surface was constructed by Frame 1, 2, and 4 and was visible from the exterior, while the non-outward opening was buried by the right copy of VP1, with a minimal size of 6–7 Å (Table 1). Multiple factors conferred the orientation of the opening as outward (denoted “1”) or non-outward (denoted “0”), including the maximum distance among the frames or the involvement of other motifs from VP3 (Figure 1F, Table 1). The analysis on the residues of the framework peptide around the pocket implied that their side-chain interactions may contribute to the size of opening (Figure S2). The displacement of Frame 1, 2, and 4 would widen the outward opening on the roof and narrow the distance of Frame 2 and 3 when transforming to an A-particle (Figure 1G, Table 1), while the bridge between Frame 1 and 2 with MOPS could stabilize the viral particle, as reported in [17]. 

### 3.2. The Orientation of the VP1-Pocket Opening Correlated with Capsid Stability 

**Figure 2:**
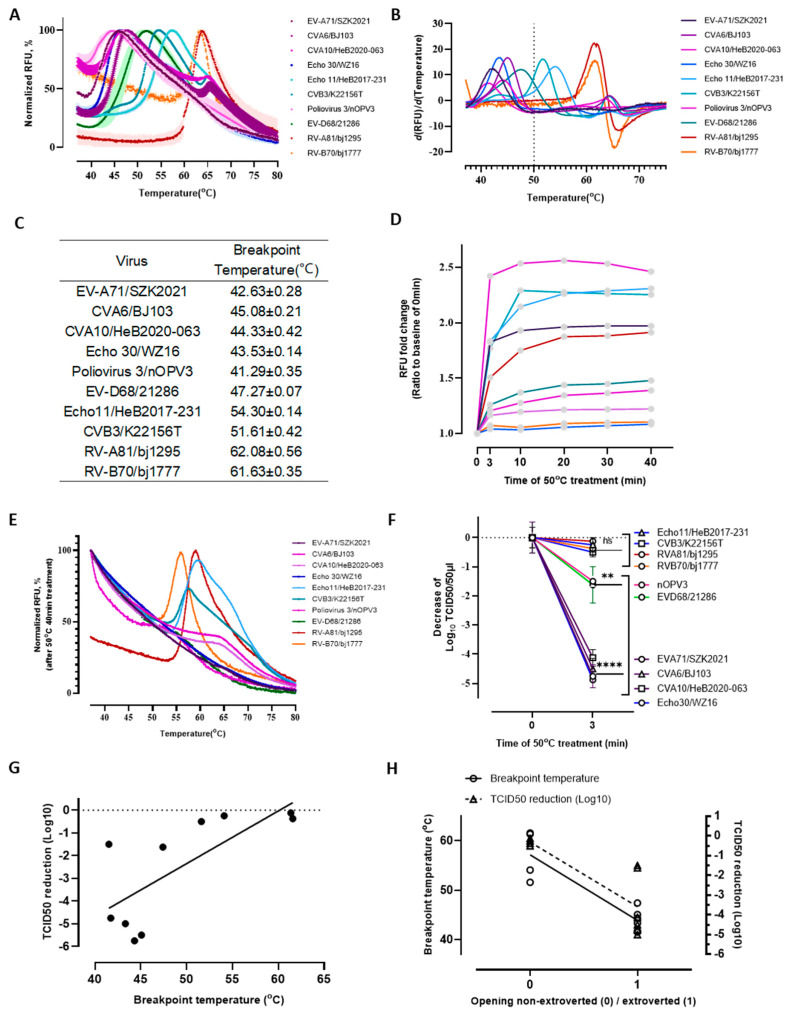
Thermostability of represented EVs and RVs. (**A**) Normalized fluorescence of Genome release of EVs and RVs. About 4 μg virus in a 20 μL reaction was set up at 37 °C for 15 min and subsequently the temperature was increased to 90 °C, with recordings of 10 points of fluorescence signal at 1 °C intervals. The experiment was repeated at least in duplicate. (**B**) First derivative of the Normalized Fluorescence identified the breakpoint temperature of the genome release. (**C**) Breakpoint temperature of EVs and RVs. (**D**) Fluorescence fold-change of genome release at 50 °C for 3, 10, 20, 30, and 40 min compared that at 0 min. The reaction set-up was the same as described previously. (**E**) The Normalized fluorescence of genome release after treatment at 50 °C for 40 min. (**F**) Infectivity difference of EVs and RVs treated at 50 °C for 3 min compared to 0 min (without 50 °C treatment). The experiment was repeated at least in triplicate. Statistical analysis was performed by one-way ANOVA with multiple comparisons. ** indicates *p* < 0.01 and **** indicates *p* < 0.0001. (**G**) Correlation of Log_10_TCID_50_ and breakpoint temperature (Pearson’s *r* = 0.77, *p* = 0.009). (**H**) Correlation of the pocket opening with breakpoint temperature (Pearson’s *r* = −0.90, *p* = 0.0004) and Log_10_TCID_50_ reduction post-50 °C treatment (Pearson’s *r* = −0.82, *p* = 0.0039) with linear regression.

An important aspect of EV viruses is their thermal stability, so we assayed the fluorescence signals of dyes bound with nuclei acids released from EVs by heating. We found that the dynamics of the genome release curve were disparate for different EVs and RVs (Figure 2A,B) and could be grouped into those releasing at temperatures less or greater than 50 °C. Although the B.T. calculated by the derivative of each curve indicating the unfolding of all particles was divergent, it was indeed unique for each virus strain or type. Ten strains highly homologous to the reference strains from PDB (Figure S3) were tested. The B.T. of EV-A71/SZK2021, CVA6/BJ103, CVA10/HeB2020-063, Echo 30/WZ16, Poliovirus 3/nOPV3, and EV-D68/21286 was 42.63 ± 0.28 °C, 45.08 ± 0.21 °C, 44.33 ± 0.42 °C, 43.53 ± 0.14 °C, 41.29 ± 0.35 °C, and 47.27 ± 0.07 °C, respectively. In contrast, the B.T. of Echo 11/HeB2017-231, CVB3/K22156 T, RV-A81/bj1295, and RV-B70/bj1777 was 54.30 ± 0.14 °C, 51.61 ± 0.42 °C, 62.08 ± 0.56 °C, and 61.63 ± 0.35 °C, respectively—4–10 °C higher than those below 50 °C (Figure 2C). 

To identify whether 50 °C could be a cut-off point for heat-resistance selection, we assayed the residual viruses after different treatment durations and collected the samples at 0, 3, 10, 20, 30, and 40 min after 50 °C treatment. Their genome release and infectivity were compared (Figure 2). Only Echo 11/HeB2017-231, CVB3/K22156T, RV-A81/bj1295, and RV-B70/bj1777 retained genome release even after 40 min treatment (Figure 2D,E), and similarities were found in their infectivity. Minor reductions in the corresponding Log_10_TCID_50_ titer were found: −0.250 ± 0.250, −0.500 ± 0.177, −0.125 ± 0.125, and −0.375 ± 0.28, respectively (Figure 2F). This reduction was closely correlated to B.T. (*r* = 0.771, *p* = 0.009) (Figure 2G).

Considering the exposed pocket factor of the outward opening might contribute to a lower B.T., we took outward or non-outward as a dichotomous variable and compared it. Notably, the opening orientation was well correlated with the B.T. (*r* = −0.90, *p* = 0.0004), as well as infectivity reduction after 50 °C treatment (*r* = −0.82, *p* = 0.0039) (Figure 2H), suggesting a possible effect of pocket opening on the capsid stability.

### 3.3. The Energy for the Pocket Framework but Not for Binding with the Pocket Factor Related to Capsid Stability

Frames 1, 2, and 3 were involved in interactions with the pocket factor, as reported previously [7,9]. We calculated the binding energy of the pocket factor using a promoter (Table S1). Consistent with previous observations, there was energy barrier for the EV conformational conversion, but the pocket factor was not sufficient to trigger the rearrangement into an A particle [5,18]. The binding energy did not show a correlation with B.T., such as in the instance of Echo 11 having a weaker binding energy of −83.39 kJ/mol, but also having a non-outward pocket opening on the wall as compared with the others, as well as a higher B.T of 54.30 ± 0.14 °C. It is likely that the binding energy indicated how strong this pocket drags the pocket factor inside; even though this bond was disrupted, the molecule would still require additional energy to expel out. 

Since the pocket factor left the roof of the pocket, to explore whether the framework peptide made a contribution to viral thermostability, we analyzed the energy of Frame 1, 2, and 4 by EvoEF2 (Table 2). The atomic forces, including Van der Waals attractive, Van der Waals repulsive, and hydrogen bond terms, showed significant correlations with B.T. (r = −0.67, 0.75, and −0.8; *p* = 0.034, 0.013, and 0.006, respectively). Frame 2 possessed part of the GH loop, which has been found to be involved in intra-protomer contacts, which restrict the conformational change and RNA ejection during uncoating [19,20]. Our findings suggested that the framework was involved in the particle interactions and maintained its stability.

## 4. Discussion

In this study, we discovered and proposed a structural framework for the VP1-pocket and grouped its openings into outward and non-outward. We explored their correlations with capsid thermostability by measuring the release of the encapsulated genome of the representative EVs and RVs. We found that the B.T. is unique for each virus strain or type, but is usually <50 °C for viruses with outward openings and >50 °C for those with non-outward openings. Thermal stability was found in those with a non-outward opening. Furthermore, the energy analysis implied that the force between Frames 1, 2, and 4 was correlated with heat-resistance, indicating the amino acid composition and opening appearance as robust predicators for virion thermostability. However, the structural framework of the VP1-pocket is not static but dynamic; more data on virus structures and molecular dynamics simulations may enhance accuracy on this framework. 

A series of particle-stabilizing contacts and their corresponding key motifs or residues have been documented; for example, the VP2 or VP3 between capsid subunits or the “pocket factor” within subunits or nectin-like binding interactions [5,8,21,22]. In our proposed framework, similar key residues or segments—such as VP1 112D and 203W, the known receptor-binding sites involved in uncoating [23,24], and Frame 2 bearing some of the GH loop—that mediated the protomer–protomer interactions [25] were also included. Additionally, the amino acids around the opening were highly variable among the EVs, implying that a complicated modulating network for capsid stability and a strategy for developing heat-resistant vaccines would require multiple mutations. Modulations on the residues that determine the opening size listed in Table 1 in our study might be an alternative option, and need further investigation.

Collectively, the data presented here support that theory the thermostability can be figured out based on the framework domain of the VP1-pocket and its openings.

## Figures and Tables

**Table 1:** Characterization of VP1-pocket opening.

Virus	Full Particle					A-Particle			
	PDB ID	Opening	Max. Distance of FR1–FR2 (Å)	Max. Distance of FR2–FR3 (Å)	Other Peptide Involved	PDB ID	Opening Change FR1/FR2	Opening Change FR2/FR3	Mainly Moved Frame
EV-A71	8E2X	1	10.4 (112D–224A)	5.8 (227N–201Y)	FR4	3J22	enlarged	NO	FR1
CVA6	7QW9	1	11.3 (108M–219C)	5.9 (221N–195Y)	FR4	5XS4	enlarged	NO	FR1
CVA10	6ACU	1	8.1 (111D–228M)	3.6 (226N–200Y)	FR4	6ACY	enlarged	NO	FR1
Echo 30	7C9S	1	8.1 (97T–211Y)	7.2 (214L–193Y)	FR4	7C9T	enlarged	Closed	FR2
Echo 11	6LA3	0	3.5 (96S–214N)	7.9 (213L–192Y)	FR4	6LAP	enlarged	Closed	FR2
CVB3	7VY0	0	2.5 (92V–213M)	7.8 (210L–189Y)	FR4	7VXL	enlarged	Closed	FR2
PV3	1PVC	1	11.4 (111T–232A)	12.0 (234T–205Y)	FR4	NA	NA	NA	NA
EV-D68	6CRR	1	10.8 (97T–212N)	4.0 (215D–193Y)	FR4	6CRS	enlarged	NO	FR2
RV-A16	1ND2	0	5.2 (96N–212N)	7.4 (212N–190Y)	FR4	NA	NA	NA	NA
RV-B14	1R09	0	8.3 (105N–216T)	9.1 (218L–197Y)	VP3-236E	NA	NA	NA	NA

Opening was denoted as “1” for outward and “0” for non-outward. NO: not observed. NA: not available. FR: Frame. Max. distance was measured structurally between the amino acid as indicated.

**Table 2:** Pocket framework peptide interaction energy (kcal/mol).

Virus	PDB ID	B.T. (°C)	Opening	vdwatt	vdwrep	electr	hb
EV-A71	8E2X	42.63	1	−41.22	2.57	−3.49	−8.2
CVA6	7QW9	45.08	1	−39.52	6.77	−0.54	−4.1
CVA10	6ACU	44.33	1	−39.54	2.34	−1.89	−5.52
Echo 30	7C9S	43.53	1	−49.38	1.18	−1.46	−7.77
Echo 11	1H8T	54.3	0	−48.67	16.62	−1.52	−10.86
CVB3	7VY0	51.61	0	−43.87	4.19	−2.63	−7.05
PV3	1PVC	41.29	1	−39.13	3.34	−1.99	−3.13
EV-D68	4WM8	47.27	1	−41.44	2.45	−1.03	−6.54
RV-A16	1ND2	62.08	0	−56.72	11.77	−3.02	−11.73
RV-B14	1R09	61.63	0	−44.42	9.68	−1.51	−10.11
Corr. with B.T.			−0.90	−0.67	0.75	−0.13	−0.80
*p* value			0.0004	0.034	0.013	0.725	0.006

Pocket Frame 1, Frame 2, and Frame 4 on the surface were calculated. B.T. Breakpoint temperature. vdwatt: Van der Waals attractive. vdwrep: Van der Waals repulsive. electr: Coulomb’s electrostatics. hb: Hydrogen Bond.

## Data Availability

The data are available in the main text or the Supplementary Information.

## Supplementary Materials

The following supporting information can be downloaded at: https://www.mdpi.com/article/10.3390/pathogens13080711/s1, Figure S1: VP1 sequences alignment of representing EVs and RVs [26]; Figure S2: Amino acids on VP1-pocket framework related to opening appearance; Figure S3: Maximum likely tree of VP1 sequences of experimental and structural enterovirus strains; Table S1: Pocket factor binding energy.

## References

[1] Jiang P., Liu Y., Ma H.-C., Paul A.V., Wimmer E. (2014). Picornavirus Morphogenesis. Microbiol. Mol. Biol. Rev..

[2] Tapparel C., Siegrist F., Petty T.J., Kaiser L. (2013). Picornavirus and enterovirus diversity with associated human diseases. Infect. Genet. Evol..

[3] Bertrand I., Schijven J.F., Sánchez G., Wyn-Jones P., Ottoson J., Morin T., Muscillo M., Verani M., Nasser A., de Roda Husman A.M. (2012). The impact of temperature on the inactivation of enteric viruses in food and water: A review. J. Appl. Microbiol..

[4] Rincón V., Rodríguez-Huete A., López-Argüello S., Ibarra-Molero B., Sanchez-Ruiz J.M., Harmsen M.M., Mateu M.G. (2014). Identification of the Structural Basis of Thermal Lability of a Virus Provides a Rationale for Improved Vaccines. Structure.

[5] Strauss M., Filman D.J., Belnap D.M., Cheng N., Noel R.T., Hogle J.M., Kirkegaard K. (2015). Nectin-Like Interactions between Poliovirus and Its Receptor Trigger Conformational Changes Associated with Cell Entry. J. Virol..

[6] Baggen J., Thibaut H.J., Strating J.R.P.M., van Kuppeveld F.J.M. (2018). The life cycle of non-polio enteroviruses and how to target it. Nat. Rev. Microbiol..

[7] Smyth M., Pettitt T., Symonds A., Martin J. (2003). Identification of the pocket factors in a picornavirus. Arch. Virol..

[8] Egorova A., Ekins S., Schmidtke M., Makarov V. (2019). Back to the future: Advances in development of broad-spectrum capsid-binding inhibitors of enteroviruses. Eur. J. Med. Chem..

[9] Liu Y., Sheng J., Fokine A., Meng G., Shin W.H., Long F., Kuhn R.J., Kihara D., Rossmann M.G. (2015). Structure and inhibition of EV-D68, a virus that causes respiratory illness in children. Science.

[10] Adeyemi O.O., Nicol C., Stonehouse N.J., Rowlands D.J., Dermody T.S. (2017). Increasing Type 1 Poliovirus Capsid Stability by Thermal Selection. J. Virol..

[11] Cao Y., Song L., Miao Z., Hu Y., Tian L., Jiang T. (2011). Improved side-chain modeling by coupling clash-detection guided iterative search with rotamer relaxation. Bioinformatics.

[12] Abraham M.J., Murtola T., Schulz R., Páll S., Smith J.C., Hess B., Lindahl E. (2015). GROMACS: High performance molecular simulations through multi-level parallelism from laptops to supercomputers. SoftwareX.

[13] Wang J., Wolf R.M., Caldwell J.W., Kollman P.A., Case D.A. (2004). Development and testing of a general amber force field. J. Comput. Chem..

[14] Kumari R., Kumar R., Lynn A. (2014). g_mmpbsa—A GROMACS tool for high-throughput MM-PBSA calculations. J. Chem. Inf. Model..

[15] Katoh K., Misawa K., Kuma K., Miyata T. (2002). MAFFT: A novel method for rapid multiple sequence alignment based on fast Fourier transform. Nucleic Acids Res..

[16] Huang X., Pearce R., Zhang Y. (2020). EvoEF2: Accurate and fast energy function for computational protein design. Bioinformatics.

[17] Carson S.D., Hafenstein S., Lee H. (2017). MOPS and coxsackievirus B3 stability. Virology.

[18] Tsang S.K., McDermott B.M., Racaniello V.R., Hogle J.M. (2001). Kinetic analysis of the effect of poliovirus receptor on viral uncoating: The receptor as a catalyst. J. Virol..

[19] Bostina M., Levy H., Filman D.J., Hogle J.M. (2011). Poliovirus RNA Is Released from the Capsid near a Twofold Symmetry Axis. J. Virol..

[20] Rey F.A., Shingler K.L., Yoder J.L., Carnegie M.S., Ashley R.E., Makhov A.M., Conway J.F., Hafenstein S. (2013). The Enterovirus 71 A-particle Forms a Gateway to Allow Genome Release: A CryoEM Study of Picornavirus Uncoating. PLoS Pathog..

[21] Khosla C., Abdelnabi R., Geraets J.A., Ma Y., Mirabelli C., Flatt J.W., Domanska A., Delang L., Jochmans D., Kumar T.A. (2019). A novel druggable interprotomer pocket in the capsid of rhino- and enteroviruses. PLoS Biol..

[22] Smith T.J., Kremer M.J., Luo M., Vriend G., Arnold E., Kamer G., Rossmann M.G., McKinlay M.A., Diana G.D., Otto M.J. (1986). The site of attachment in human rhinovirus 14 for antiviral agents that inhibit uncoating. Science.

[23] Wang X., Peng W., Ren J., Hu Z., Xu J., Lou Z., Li X., Yin W., Shen X., Porta C. (2012). A sensor-adaptor mechanism for enterovirus uncoating from structures of EV71. Nat. Struct. Mol. Biol..

[24] He X.L., Du L.F., Zhang J., Liang Y., Wu Y.D., Su J.G., Li Q.M. (2021). The functional motions and related key residues behind the uncoating of coxsackievirus A16. Proteins.

[25] Ross C., Knox C., Tastan Bishop Ö. (2017). Interacting motif networks located in hotspots associated with RNA release are conserved in Enterovirus capsids. FEBS Lett..

[26] Robert X., Gouet P. (2014). Deciphering key features in protein structures with the new ENDscript server. Nucleic Acids Res..

