# Supplementary material for: The Structural Framework and Opening Appearance of the VP1-Pocket of Enteroviruses Correlated with Viral Thermostability
Source: Pathogens. 2024 Aug 22;13(8):711. doi: 10.3390/pathogens13080711 (PMC11357065; doi:10.3390/pathogens13080711)
Supplement: Supplementary file 1 [file pathogens-13-00711-s001.zip › pathogens-3122153-supplementary.pdf]

Supplementary Information

The structural framework and opening appearance of VP1-pocket of enteroviruses correlated to viral thermostability

Lin et al.

This file contains Supplementary Figures S1, Figures S2, Figures S3, Table S1.

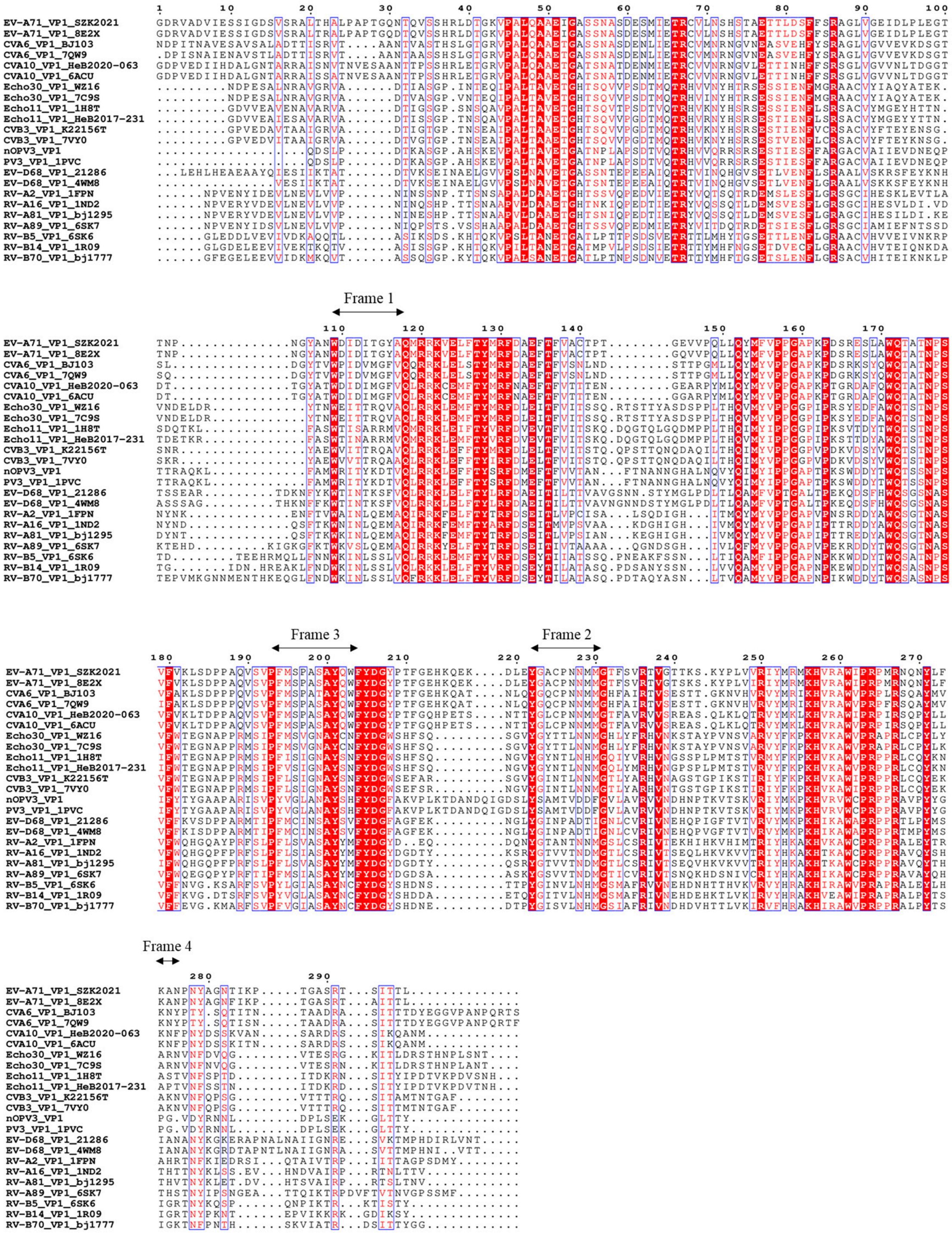

Figure S1. VP1 sequences alignment of representing EVs and RVs.

Sequences were aligned using EScript 3.0 [1].Frame 1, 2, 3 and 4 related pocket opening were indicated with double arrow.

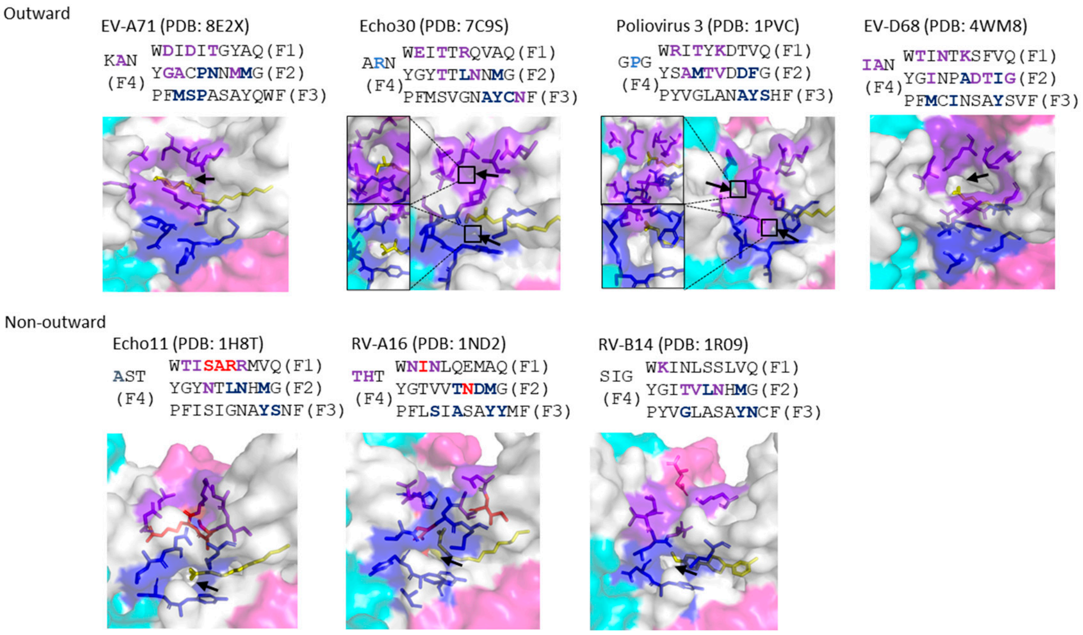

Figure S2. Amino acids on VP1- pocket framework related to opening appearance.

Amino acid (aa) of the framework peptide around the outward (up) and non-outward (down) opening. The black arrow indicated the opening forming. The pocket factor was colored in yellow, and the purple and blue indicated the aa related to the outward and non-outward opening, respectively. The aa in red indicate these aas play the role to close the outward opening. VP1, VP2 and VP3 were colored in white, cyan and magenta, respectively.

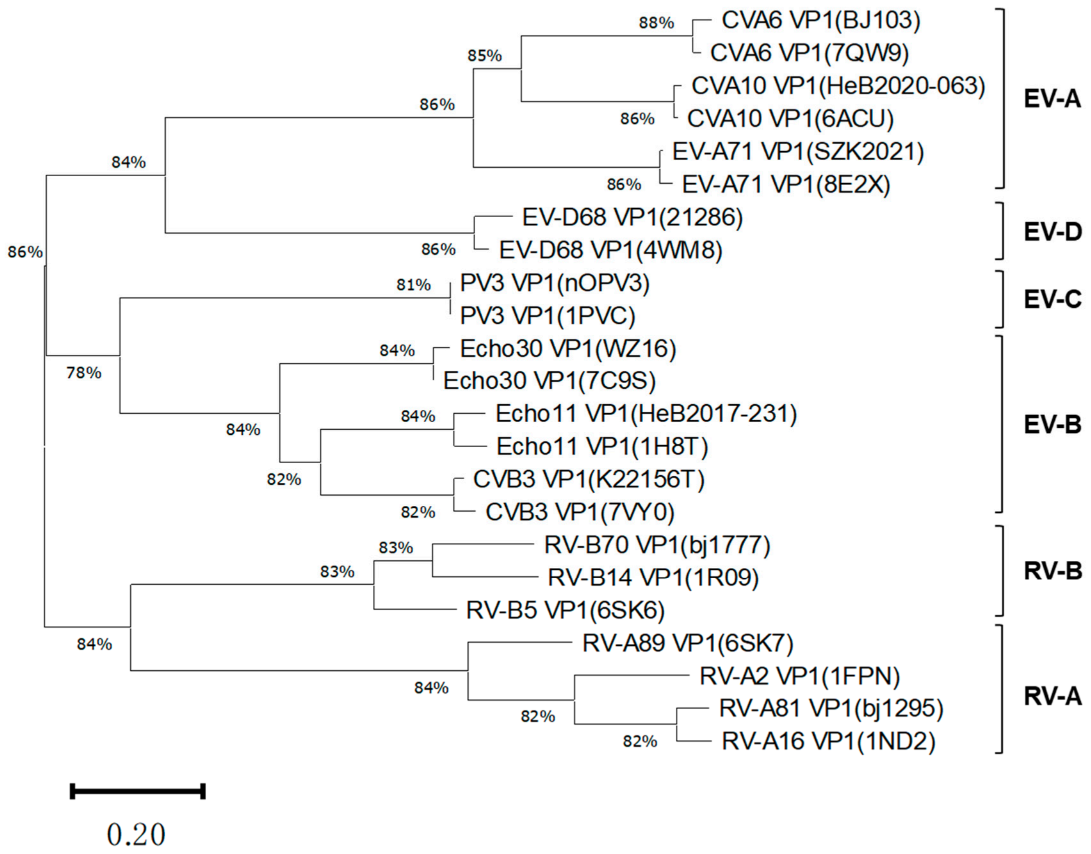

Figure S3. Maximum likely tree of VP1 sequences of experimental and structural enterovirus strains.

Table S1. Pocket factor binding energy (kJ/mol).

Virus	PDB ID	B.T.	Binding energy	SASA energy	Polar solvation energy	Electrostatic energy	van der Waal energy
EV-A71	8E2X	42.63	-139.541	-25.685	110.954	-4.501	-220.309
CVA6	7QW9	45.08	-158.786	-21.999	96.882	-0.750	-232.919
CVA10	6ACU	44.33	-189.579	-23.953	115.955	-56.232	-225.349
Echo 30	7C9S	43.53	-155.680	-24.225	131.093	-23.399	-239.149
Echo 11	1H8T	54.30	-83.388	-18.216	84.882	9.640	-159.694
CVB3	7VY0	51.61	-125.894	-22.057	82.431	-7.909	-178.359
PV3	1PVC	41.29	-161.874	-23.604	123.674	-48.753	-213.191
EV-D68	4WM8	47.27	-131.672	-14.669	121.065	-108.663	-129.405
RV-A16	1ND2	62.08	-180.674	-19.300	87.022	-68.065	-180.331
RV-B14	1R09	61.63	-125.150	-19.118	103.800	-29.125	-180.707
**Corr. with B.T.**		/	**0.258**	/	/	/	/
**P value**	/	/	**0.471**	/	/	/	/

SASA: solvent accessible surface

Supplementary Reference

[1] X. Robert, P. Gouet, Deciphering key features in protein structures with the new ENDscript server, Nucleic Acids Research 42 (2014) W320-W324. 10.1093/nar/gku316.
